# Analysis of genetic parameters and genetic trends for early growth and reproductive traits of Doyogena sheep managed under community-based breeding program

**DOI:** 10.1016/j.heliyon.2022.e09749

**Published:** 2022-06-18

**Authors:** Kebede Habtegiorgis, Aynalem Haile, Tesfaye Getachew, Manzoor Ahmed Kirmani, Deribe Gemiyo

**Affiliations:** a Southern Agricultural Research Institute, Areka Agricultural Research Center, P.O. Box 79, Areka, Ethiopia; b Jmma University College of Agriculture and Veterinary Medicine, P.O. Box, 307, Jimma, Ethiopia; c International Center for Agricultural Research in the Dry Areas, P.O. Box. 5689, Addis Ababa, Ethiopia

**Keywords:** Doyogena sheep, Community based breeding, Heritability, Genetic trend

## Abstract

This study aims to estimate genetic parameters and genetic trends for early growth and reproductive traits of Doyogena sheep. Data used in the study were collected over 6 years (2013–2018). Studied traits were birth weight (BWT), weaning weight (WWT), 6-month weight (SMWT), average daily gains from birth to weaning (ADG0-3), average daily gains from weaning to 6-month age (ADG3-6), average daily gain from birth to 6-month age (ADG0-6), litter size (LS), lambing interval (LI), age at first lambing (AFL), and annual reproductive rate (ARR). (Co) variance components and genetic parameters were estimated using restricted maximum likelihood (REML). The analyses were carried out using WOMBAT program. Univariate analysis was applied to estimate genetic parameters. Six different animal models were fitted by including or excluding maternal effects. The direct heritability estimates for BWT, WWT, SMWT, ADG0-3, ADG3-6 and ADG0-6 were 0.33 ± 0.06, 0.31 ± 0.06, 0.14 ± 0.06, 0.13 ± 0.04, 0.11 ± 0.07, and 0.02 ± 0.05 respectively. Direct heritability for LS, LI, and AFL were 0.28 ± 0.12, 0.20 ± 0.5, and 0.001 ± 0.3, respectively. The maternal heritability estimates for BWT, WWT, and LS were 0.24 ± 0.12, 0.60 ± 0.07, and 0.24 ± 0.08, respectively. The genetic correlation between BWT with WWT and BWT with SMWT were 0.21 ± 0.07 and 0.21 ± 0.09, respectively. Genetic progress for most of the studied traits has shown promising improvements. Thus, continuation of selection, therefore, suggested for more improvements in the performance of Doyogena sheep. Direct heritability estimates decrease as lamb age increases and selection based on earlier body weight will be more efficient.

## 1 Introduction

Small ruminants, particularly native breed kinds play a significant role in the livelihoods of a considerable part of human population in the tropics from socio-economic aspects (Ahsani et al., 2010; Mohammadabadi 2021; Masoudzadeh et al., 2020a). Thus, combined trials with emphasis on administration and genetic progress to improve animal outputs are of decisive significance (Masoudzadeh et al., 2020b; Mohammadabadi et al., 2017). Economical and biological efficiency of sheep production enterprises generally improves by increasing productivity and reproductive performance of ewes (Mohammadabadi 2016; Amiri Roudbar et al., 2017, 2018; Ghotbaldini et al., 2019; Mohammadabadi et al., 2021).

Community-based breeding programs (CBBPs) have recently attracted global interest as genetic improvement strategies in low input systems (Lamuno et al., 2018; Haile et al., 2019). CBBP has been designed to ensure the involvement of farmers (target groups) in all steps of the breeding program (Mueller et al., 2015). In Ethiopia, in 2012, the International Center for Agricultural Research in the Dry Areas (ICARDA), in partnership with the Southern Agricultural Research Institute (SARI), Areka Agricultural Research Centre (AARC) adopted CBBP in the Doyogena district to improve Doyogena sheep. Doyogena sheep are among the potential breeds of the country with better market preferences in the local market and Addis Ababa. The sheep have attractive morphological features with great potential for twining and fattening.

The potential genetic improvement of traits of interest was largely dependent on its heritability and genetic relationship among the traits of economic importance upon which selection may be applied. Information on heritability is essential for planning efficient breeding programs, and for prediction of response to selection (Falconer and Mackay, 1996). According to Berkana (2019), evaluation of any designed genetic improvement program is fundamental either to optimize the program if the designed improvement program is progressing towards the set goals or redesign other alternatives if it fails or deviates from the preset goals. Moreover, evaluation of genetic trends gives an indication of the genetic direction of the breed as well as the rate of genetic improvement from the time of application of the breeding program (Mallick et al., 2016). However, genetic studies of productive traits in sheep in low input systems are scarce due to lack of recorded data (Aguirre et al., 2017).

The present study has been planned to estimate the genetic parameters and trends for growth and reproductive traits and generate information for the optimization of the ongoing CBBP.

## 2 Material and methods

### 2.1 Description of the study area

The study area (Doyogena sheep CBBP) is found in Kembata Tembaro Zone of southern Ethiopia at a distance of 258 km to the Southwest of Addis Ababa (national capital). The district is located between 7°20′ N latitude and 37°50' E longitude. Altitude ranges from 1900 to 2800 m. a.s.l. The average annual rainfall of the district is 1221 mm.

### 2.2 Breeding program description and animal management

Animals were identified by plastic ear tags. Enumerators were employed for routine animal identification, data recording, and follow-up. Enumerators use herd books for data recording. Selection of breeding rams takes place on a programmed date, twice a year. Researchers identify the candidate breeding rams by estimating best linear unbiased prediction (BLUP) breeding values for selection criteria traits using the performance and pedigree data recorded by the enumerators. Lambs wean at the age of 90 days (three-months age). Then the candidate rams pre-selection and ranking take place based on weaning weight. In the second stage-breeding ram ranking was carried out based on SMWT estimated breeding values (EBVs). Top (10% of the candidates) breeding rams were retained for breeding to be used in the community flock while the next best (positive EBVs) were sold for breeding purposes to other communities.

Culled males (negative EBVs) were either castrated or marketed to prevent unwanted mating (Haile et al., 2019). Selected best breeding rams usually serve not more than one year in the community flock. After one year of service, the breeding rams were sold to another area of the region.

The main feed sources for animals included *Enset* or false banana (*Ensete ventricosum* (Welw. Chessman) products of *Amicho* (fresh parts of false banana could be cooked like potatoes), corm (swollen underground parts of false banana), crop residue, improved forage/grass, kitchen leftover, and purchased concentrates. Flocks graze with tethering on the small private land. Free veterinary service was provided for CBBP participant farmers by ICARDA and SARI.

### 2.3 Data sets

The empirical data for the study were obtained from five ongoing breeder cooperatives. The performance data along with pedigree information were maintained in the data-recording book of individual breeder cooperatives. The data routinely collected by the enumerators were recorded at the time of the event. BWT was recorded within 24 h of lambing; WWT was taken at 90 days of age, and 6-month weight was taken at the age of 180 days. The average daily body weight gains from birth to weaning age; weaning to six months of age, and from birth to six months of age have been estimated as under:

(1) $$\text{Average daily body weight }\left(\text{BW}\right)\text{gain up to weaning age }\left(\text{g}\right)=\frac{\text{WWT}-\text{BWT}}{90}\text{∗}1000$$

(2) $$\text{Average daily BW gain from weaning up to }6\text{ months age }\left(\text{g}\right)=\frac{\text{SMWT}-\text{WWT}}{90}\text{∗}1000$$

(3) $$\text{Average daily BW from birth to }6\text{ month age }\left(\text{g}\right)=\frac{\text{SMWT}-\text{BWT}}{180}\text{∗}1000$$

Where:

BWT = Birth weight,
WWT = Three month weight/weaning weight at 90 days,
SMWT = 6-month weight at 180 days

The annual reproductive rate, which is the number of lambs born per breeding ewes per year has been estimated according to Shigdaf (2013) as under:

(4) ARR=ALS ∗ 365÷LI

Where:

ALS = Average litter size;
LI = Lambing interval;
365 = Days of one year;

### 2.4 Statistical analysis

The variance components and resulting genetic parameters were estimated on a model-fitting effect of parity, year of birth/year of lambing, season of birth/season of lambing, type of birth, sex, and sites (cooperatives) as fixed factors. Based on the first performed analysis of variance using SAS (SAS, 2009), significant fixed effects were identified to be included in the models. Then, significant fixed effects were fitted in the subsequent models for estimating genetic parameters. Genetic parameters for reproductive traits were estimated by WOMBAT software (Meyer, 2012). Univariate animal models were fitted to estimate genetic parameters. Similarly, bivariate, trivariate, and multivariate analyses were applied for estimation of correlations. Direct additive and maternal additive genetic effects with or without a covariance between them, and maternal permanent environmental effects were tested for all traits in different combinations to yield six models. The six models were as follows:

**Table:** 

**(Co) variance components**	**models**	
$\text{y}=\text{xb}+{\text{z}}_{1}\text{a}+\text{e}\;$	(model 1)	(5)
$\text{y}=\text{xb}+{\text{z}}_{1}\text{a}+{\text{z}}_{2}\text{c}+\text{e}$	(model 2)	(6)
$\text{y}=\text{xb}+{\text{z}}_{1}\text{a}+{\text{z}}_{2}\text{m}+\text{e with cov}\left(\text{a},\text{m}\right)=0\;$	(model 3)	(7)
$\text{y}=\text{xb}+{\text{z}}_{1\text{a}}+{\text{z}}_{2}\text{m}+\text{e},\text{with cov}\left(\text{a},\text{m}\right)=\text{Aσam}$	(model 4)	(8)
$\text{y}=\text{xb}+{\text{z}}_{1}\text{a}+{\text{z}}_{2}\text{m}+{\text{z}}_{3}\text{c}+\text{e},\text{with cov}\left(\text{a},\text{m}\right)=0\;$	(model 5)	(9)
$\text{y}=\text{xb}+{\text{z}}_{1}\text{a}+{\text{z}}_{2}\text{m}+{\text{z}}_{3}\text{c}+\text{e};\text{with cov}\left(\text{a},\text{m}\right)=\text{Aσam}$	(model 6)	(10)

Where:

y = vector of observed traits;
b, a, m, c = Vectors of fixed effects, direct additive genetic effects,
Maternal additive genetic effects, maternal permanent environmental effects and
Vector respectively. e = residual effects

X, Z,_1_, Z_2,_ and Z_3_ = Incidence matrices, respectively relating fixed effects, direct additive genetic effects, maternal additive genetic effects, and maternal permanent effects to y; All components with the phenotypic variance (σ2p) being the sum of σ2a, σ2m, σam, σ2c, and σ2e, were derived at convergence. Depending on the model, we computed

(11) $$\text{Direct heritability as; }h^{2}=\frac{\sigma^{2}a}{\sigma^{2}p}$$

(12) $$\text{Maternal heritability; }m^{2}=\frac{\sigma^{2}m}{\sigma^{2}p}\text{ and;}$$

the direct-maternal covariance as proportion of phenotypic variance; (c_am_ = σ_am_/σ^2^p), with a corresponding estimate of the direct-maternal correlation [r_am_ = c_am_/(σ^2^_a_ ∗σ^2^_m_)]. Similarly maternal environmental variance ratio was estimated by the maternal permanent environmental variance as a proportion of σ^2^p (p^2^ = σ^2^c/σ^2^p). The genetic correlation between direct and maternal genetic effects (r_am_) is estimated as the ratio of the estimates of the σ_am_ to the product of the square roots of the estimates of σ^2^_a_ and σ^2^_m_ (Meyer (2007).

(13) $$\;r_{am}=\frac{{\text{σ}}_{am}}{\sqrt{\text{σ}a^{2}\text{∗σ}m^{2}}}$$

Total heritability (h^2^t) was calculated according to the following equation (Willham, 1972).

(14) $$h^{2}t=\frac{\sigma^{2}+0.5\sigma m^{2}+1.5\text{σam}}{\text{σ}{\text{p}}^{2}}$$

To determine the most appropriate model, likelihood ratio tests (logL) were used for each trait. An effect was considered to have significant influence, when its inclusion caused significant increase in log-likelihood, compared to the model in which it was ignored. When log-likelihoods did not differ significantly (P > 0.05), the model that has fewer parameters was selected as the most appropriate model. All models included direct additive genetic effect and this was the only random factor in Model 1. Model 2 included the maternal permanent environmental effect, fitted as an additional random effect. Model 3 included an additive maternal effect fitted as second random effect. Model 4 was the same as Model 3 but allowed for a direct maternal covariance Cov_(a,m)_. Model 5 and Model 6 included additive maternal and maternal permanent environmental effects, ignoring and fitting, respectively, direct-maternal covariance.

The genetic trends were estimated by the weighted regression of the average breeding value of the animals on the year of birth or year of lambing. These procedures were carried out with statistical program R core software (R, 2018) and MS-excel. Genetic change for the traits over the selection period was calculated by subtracting the mean of the estimated breeding values at the beginning of the CBBP from the mean of the EBV at the time of this study’s 2018-selection year.

## 3 Results and discussion

### 3.1 Genetic parameter estimation

#### 3.1.1 Data and pedigree structure

In Table 1 the structure of pedigree has been presented.

**Table 1:** Characteristics of the pedigree structure for studied traits.

Item	Numbers
Number of animals	4497
Number of records	2990
Number of dams	899
Number of sires	326
Number of animals with unknown sire	1238
Number of animals with unknown dam	1465
Number of animals with both parents unknown	992
Number of animals with records and both parents unknown	72

## 4 Inbreeding

**Figure 1:**
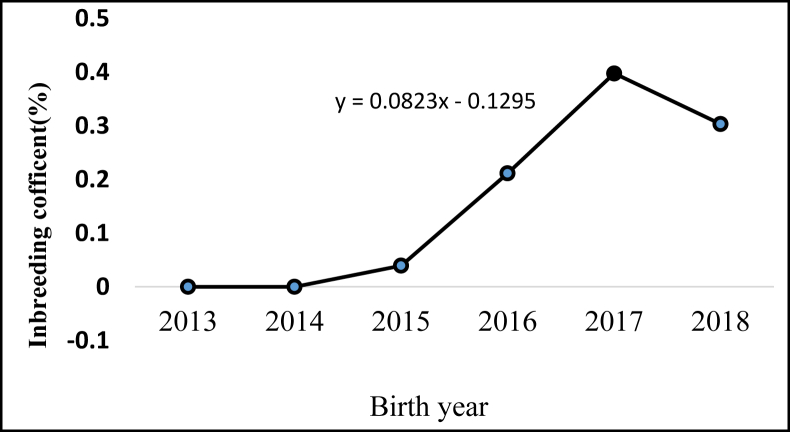
Annual mean of inbreeding.

The coefficient of inbreeding (F) showed an increasing trend within the 6-year selection period (Figure 1). The coefficient of inbreeding was assumed to be zero until the year 2014; afterward, it increased with the selection years. At the time of this study (2018 selection year) coefficient of inbreeding was 0.30% with an average annual inbreeding trend of 0.08%. The proportions of inbred animals from 4497-studied animals were 37 animals. The most likely reason for this inbreeding increment could be the selection of superior breeding rams without seeing their detailed pedigree. FAO (2010) suggested that the inbreeding rate must be maintained lower the range of 0.5–1% per year to avoid risks of genetic disorders and inbreeding depression. The inbreeding coefficient obtained for Doyogena sheep was considered as an acceptable percentage, however, F is in increasing trend, and thus consideration should be given during allocation of breeding rams. Negussie et al. (2002) reported 19 years (1978–1997) inbreeding coefficient of 0.78% with an annual trend of 0.07% for Horro sheep on the station management system. Gizaw et al. (2013) reported F for Menz sheep after 10-years selection was 1.7% with 0.17% increment per generation, which is higher than the presently estimated inbreeding coefficient. Generally, inbreeding leads to a reduction in additive genetic variance and heritability (Falconer and Mackay, 1996).

## 5 Genetic parameter estimates for growth traits

Based on the appropriate models the estimates of direct heritability (h^2^a) for BWT, WWT, and SMWT were 0.33 ± 0.06, 0.31 ± 0.06, and 0.14 ± 0.06, respectively (Table 2). Except for the moderate heritability estimate for SMWT, which reflects less genetic variation among lambs, the estimates of h^2^a for BWT and WWT fall within the range of values reported in the high heritability value. The present estimate of direct heritability for BWT (0.33 ± 0.06) was found in the range reported by Yacob (2008) h^2^a estimated for Afar sheep (0.1–0.38) and blackhead somalin (BHS) sheep (0.2–0.58) using univariate analysis. Gizaw et al. (2007) estimated a very high estimated h^2^a of 0.46 from a multi-trait animal model for Menz sheep, while Abegaz et al. (2002) estimated h^2^a of 0.20 ± 0.05 for Horro sheep using the same model and reported lower estimates than the currently estimated h^2^a for Doyogena sheep. The current result was higher than the estimate of Assan et al. (2002) for Sabi sheep (0.28), El Fadili et al. (2000) for Moroccan Timahdit sheep (0.18), Gizaw et al. (2014b) for Menz sheep (0.019 ± 0.036) using multi-trait individual animal model analysis and Haile et al. (2020) for Bonga (0.29 ± 0.047), Horro (0.16 ± 0.040) and Menz sheep (0.07 ± 0.027) by fitting univariate animal model.

**Table 2:** Estimates of (co) variance components and genetic parameters for growth traits.

Birth weights							
Models	Model 1	Model 2	Model 3	Model 4	Model 5	Model 6	
${{\text{σ}}^{2}}_{a}$	0.14	0.07	0.069	0.095	0.073	0.09	
${{\text{σ}}^{2}}_{c}$	-	0.08	-	-	0.071	0.06	
${{\text{σ}}^{2}}_{m}$	-	-	0.08	0.137	0.015	0.08	
${\text{σ}}_{am}$	-	-	-	-0.06	-	-0.05	
${{\text{σ}}^{2}}_{e}$	0.13	0.12	0.12	0.11	0.12	0.12	
${{\text{σ}}^{2}}_{p}$	0.28	0.28	0.28	0.28	0.28	0.28	
$h^{2}a\pm S.E$	0.51 ± 0.04	0.26 ± 0.04	0.24 ± 0.04	0.33 ± 0.0	0.25 ± 0.05	0.33 ± 0.06	
$c^{2}\pm S.E$	-	0.3 ± 0.02	-	-	0.25 ± 0.08	0.20 ± 0.09	
$h^{2}m\pm S.E$	-	-	0.30 ± 0.02	0.48 ± 0.0	0.05 ± 0.08	0.24 ± 0.12	
$r_{am}\pm SE$	-	-	-	-0.53 ± 0.1	-	-0.61 ± 0.15	
h^2^t	0.51	0.26	0.39	0.25	0.28	0.21	

Weaning weight							
${{\text{σ}}^{2}}_{a}$	2.29	2.26	1.1034	**2**	1.10	2.00	
${{\text{σ}}^{2}}_{c}$	-	0.86	-	**-**	1.00	0.79	
${{\text{σ}}^{2}}_{m}$	-	-	2.03	**3.85**	1.02	3.05	
${\text{σ}}_{am}$	-	-	-	**-2.1**	-	-2.2	
${{\text{σ}}^{2}}_{e}$	3.99	3.22	3.24	**2.72**	3.25	2.75	
${{\text{σ}}^{2}}_{p}$	6.29	6.33	6.38	**6.35**	6.38	6.35	
$h^{2}a\pm S.E$	0.36 ± 0.05	0.35 ± 0.05	0.17 ± 0.04	**0.31 ± 0.06**	0.17 ± 0.04	0.31 ± 0.06	
$c^{2}\pm S.E$	-	0.14 ± 0.02	-	**-**	0.16 ± 0.03	0.22 ± 0.08	
$h^{2}m\pm S.E$	-	-	0.32 ± 0.03	**0.60 ± 0.07**	0.16 ± 0.0	0.39 ± 0.01	
$r_{am}\pm SE$	-	-	-	**-0.81 ± 0.11**	-		-0.99
h^2^t	0.36	0.35	0.33	**0.12**	0.25		0.04

6-month weight							
${{\text{σ}}^{2}}_{a}$	3.6	**1.30**	1.16	1.93	1.28		1.82
${{\text{σ}}^{2}}_{c}$	-	**3.01**	-	-	2.77		4.9
${{\text{σ}}^{2}}_{m}$	-	**-**	3.003	4.20	0.25		2.44
${\text{σ}}_{am}$	-	**-**	-	-1.50	-		-1.14
${{\text{σ}}^{2}}_{e}$	5.85	**5.11**	5.28	4.88	5.12		4.89
${{\text{σ}}^{2}}_{p}$	9.5078	**9.44**	9.44	9.44	9.43		9.43
$h^{2}a\pm S.E$	0.38 ± 0.06	**0.13 ± 0.06**	0.12 ± 0.06	0.20 ± 0.083	0.14 ± 0.06		0.19 ± 0.08
$c^{2}\pm S.E$	-	**0.32 ± 0.04**	-	-	0.29 ± 0.20		0.14 ± 0.22
$h^{2}m\pm S.E$	-	**-**	0.31 ± 0.04	0.44 ± 0.11	0.02 ± 0.20		0.25 ± 0.29
$r_{am}\pm SE$	-	**-**	-	-0.55 ± 0.26	-		-0.54 ± 0.38
h^2^t	0.38	**0.14**	0.28	0.19	0.15		0.14

σ^2^a = direct additive genetic variance; σ^2^c = maternal permanent environmental variance; σ^2^m = maternal additive genetic variance; σ_am_ = additive and maternal additive genetic covariance, σ^2^e = residual variance, σ^2^p = phenotypic variance, h^2^a = direct heritability c^2^ = ratio maternal permanent environmental variance to phenotypic variance, h^2^m = maternal heritability; r_am_ = correlation between direct maternal additive genetic effects, h^2^t = total heritability and SE = standard error.

The estimate of h^2^a for WWT (0.31 ± 0.06) was also found in the estimated range reported by Yacob (2008) for Afar sheep (0.11–0.37) and BHS sheep (0.00–0.29) but lower than the estimate for Menz sheep (0.46) by Gizaw et al. (2007). The estimate h^2^a for WWT by Abegaz et al. (2002) for Horro sheep (0.16 ± 0.05) and Gizaw et al. (2014b) for Menz sheep (0.19) were lower than the present estimate.

The estimate of h^2^a for SMWT 0.14 ± 0.06 was found in the range of h^2^a estimated for Afar sheep (0.11–0.37) and BHS (0–0.29), while, the report of Gizaw et al. (2014b) for Menz sheep (0.46) were much higher than the present estimate. Abegaz et al. (2002) estimated 0.18 ± 0.05 of h^2^a for Horro sheep, which is lower than the current estimate. From the genetic point of view, h^2^a estimated for BWT and WWT indicated that high variation within the breed would be a greater opportunity for selection response during genetic improvement through selection for these traits. Moreover, WWT will be the best criterion for selection to increase the pre-weaning growth rate because, selection based on BWT, which has the highest heritability could cause dystocia. However, the confounding effect of direct genetic and maternal genetic effects needs care.

The permanent maternal environmental effect (c^2^) for BWT was moderate in this study (0.20 ± 0.09). This indicates the importance of maternal environment and care at the birth of lambs. The current estimate is similar to the findings of Gowane et al. (2010) in Bharat Merino sheep (0.19 ± 0.02). For SMWT trait, the maternal environmental effect is more important than maternal genetic effect. Venkataramanan (2013) reported a similar finding for Nilagiri and Sandyno Indian sheep breeds. The result suggested that, even if maternal effects tend to diminish with age, some adult traits would nevertheless contain this source of variation. The current finding indicated that maternal heritability (h^2^m) is an important ratio for BWT, WWT, and the estimates were 0.24 ± 0.12 and 0.60 ± 0.07 respectively.

In the current study, for BWT, h^2^m estimate was in the range estimated (0.06–0.46) for blackhead somilan sheep (BHS) by Yacob (2008). Abegaz (2002); Assan et al. (2002) and Yacob (2008) estimated h^2^m of 0.12 ± 0.2, 0.24, and 0.02–0.21 for Horro sheep, Sabi sheep, and Afar sheep respectively and these all value are lower than the present estimate. However, Mousa et al. (2013) reported higher estimated values of h^2^m for Moroccoian Timahdit sheep (0.59). The present estimated maternal heritability of 0.6 ± 0.07 for WWT was higher than the above-mentioned maternal heritability estimates. High and negative additive maternal genetic correlation estimates were observed (Table 3) for BWT (−0.61 ± 0.15) and WWT (−0.81 ± 0.11) traits. Similar results were summarized by Safari and Fogarty (2003) for a wide range of sheep breeds. The correlation estimates between direct additive and maternal genetic effect (r_am_) for both the traits become negative means improvement in one will result in a reduction of another. The result might be due to the structure of the data set used in the analysis i.e. the number of generations the animals were measured both directly and as dams were limited causing lack of large pedigree. The estimates of total heritability (h^2^t) for BWT, WWT, and SMWT were 0.21, 0.12, and 0.14, respectively. The h^2^t estimated by Abegaz (2002) for Horro sheep for BWT, WWT, and SMWT were 0.14, 0.12, and 0.21, respectively showing little increment across lamb age, which is slightly in contrast with the present results. The result indicated that maternal effects were important for weights until about 6 months of age.

**Table 3:** Co-variance components and genetic parameter estimates for daily weight gain traits.

Average daily gain from birth to weaning (ADG0-3)						
Models	Model 1	Model 2	Model 3	Model 4	Model 5	Model 6
${{\text{σ}}^{2}}_{a}$	230.49	**114.2**	115.16	152.39	114.2	144.1
${{\text{σ}}^{2}}_{c}$	-	**179**	-	-	179	198.89
${{\text{σ}}^{2}}_{m}$	-	**-**	179.71	297.48	0.007	83.796
${\text{σ}}_{am}$	-	**-**	-	-131.57	-	-109.88
${{\text{σ}}^{2}}_{e}$	648.09	**587**	590.35	565.52	587	570.03
${{\text{σ}}^{2}}_{p}$	878	**880**	885.22	883.82	880.4	880.03
$h^{2}a\pm S.E$	0.26 ± 0.05	**0.12 ± 0.04**	0.13 ± 0.04	0.17 ± 0.05	0.13 ± 0.04	0.16 ± 0.05
$c^{2}\pm S.E$	-	**0.21 ± 0.03**	-	-	0.20 ± 0.12	0.22 ± 0.13
$h^{2}m\pm S.E$	-	**-**	0.20 ± 0.03	0.31 ± 0.08	0.±0.125	0.09 ± 0.16
$r_{am}\pm SE$	-	**-**	-	-0.6 ± 0.2	-	-0.84 ± 0.5
h^2^t	0.26	**0.13**	0.23	0.11	0.12	0.05
Average daily gain from weaning to 6 months (ADG3-6)						
${{\text{σ}}^{2}}_{a}$	75.688	**163.72**	162.99	177.9	163.68	182.4
${{\text{σ}}^{2}}_{c}$	-	**86.654**	-	-	137.3	155.82
${{\text{σ}}^{2}}_{m}$	-	**-**	116.7	147.02	0.01	5.03
${\text{σ}}_{am}$	-	**-**	-	-34	-	-30
${{\text{σ}}^{2}}_{e}$	255.84	**237.46**	1147	1137	1127	1115.4
${{\text{σ}}^{2}}_{p}$	331.53	**1428**	1427	1427	1428	1428.5
$h^{2}a\pm S.E$	0.22 ± 0.07	**0.11 ± 0.07**	0.11 ± 0.07	0.13 ± 0.08	0.11 ± 0.07	0.13 ± 0.08
$c^{2}\pm S.E$	-	**0.09 ± 0.04**	-	-	0.09 ± 0.17	0.10 ± 0.19
$h^{2}m\pm S.E$	-	**-**	0.08 ± 0.048	0.10 ± 0.11	0.00 ± 0.17	0.004 ± 0.2
$r_{am}\pm SE$	-	**-**	-	-0.21 ± 0.8	-	-0.99 ± 0.00
h^2^t	0.23	**0.11**	0.15	0.14	0.11	0.10
Average daily weight gain from birth to 6 months (ADG0-6)						
${{\text{σ}}^{2}}_{a}$	75.6880	**7.6410**	6.1339	24.8220	6.8702	13.1730
${{\text{σ}}^{2}}_{c}$	-	**86.6540**	-	-	85.7540	87.0010
${{\text{σ}}^{2}}_{m}$	-	**-**	77.9750	132.430	0.0010	4.5529
${\text{σ}}_{am}$	-	**-**	-	-57.3320	-	-7.7418
${{\text{σ}}^{2}}_{e}$	255.84	**237.4600**	247.2400	232.000	239.0000	235.2000
${{\text{σ}}^{2}}_{p}$	331.530	**331.7600**	331.3500	331.920	331.6300	332.1900
$h^{2}a\pm S.E$	0.2 ± 0.07	**0.02 ± 0.05**	0.02 ± 0.05	0.07 ± 0.07	0.02 ± 0.06	0.04 ± 0.07
$c^{2}\pm S.E$	-	**0.26 ± 0.04**	-	-	0.25 ± 0.1	0.26 ± 0.23
$h^{2}m\pm S.E$	-	**-**	0.23 ± 0.04	0.39 ± 0.12	0 ± 0.193	0.014 ± 0.3
$r_{am}\pm SE$	-	**-**	-	-0.99 ± 0.5	-	-1.0000
h^2^t	0.23	**0.023**	0.14	0.015	0.02	0.011

σ^2^a = direct additive genetic variance; σ^2^c = maternal permanent environmental variance;σ^2^m = maternal additive genetic variance; σ_am_ = additive and maternal additive genetic covariance, σ^2^e = residual variance, σ^2^p = phenotypic variance, h^2^a = direct heritability c^2^a = ratio maternal permanent environmental variance to phenotypic variance, h^2^m = maternal heritability; r_am_ = correlation between direct maternal additive genetic effects, h^2^t = total heritability and SE = standard error.

Based on the best-fitted models, the estimated h^2^a for ADG0-3, ADG3-6 and ADG0-6 were 0.12 ± 0.04, 0.11 ± 0.07, and 0.02 ± 0.05 respectively (Table 3). The estimate indicated that the inclusion of maternal permanent environmental effects in the analyses could improve the models for daily weight gain traits. The fractions of maternal permanent environmental variance are highly reflected for all considered average daily weight gain traits.

The estimate indicates variance due to permanent maternal environmental effects (c^2^) for ADG0-3 (0.21 ± 0.03) and ADG0-6 (0.26 ± 0.04) have been found significantly higher than later age daily weight gain traits of ADG3-6 (0.09 ± 0.04). It decreases with increasing lamb age. This could be due to the influences of feeding levels at the later age of the lambs and the maternal behavior of the dam, especially for pre-weaning growth traits in the lambs. The value of maternal permanent environmental variance in model (2) for this trait is not significantly different from other models' values. The result is also found in the range reported by Yacob (2008) for Afar and BHS sheep and lower than the report of Radwan and Shalaby (2017) and Matika (2001) for Rahmani and Sabi sheep, respectively. The estimate of h^2^a for ADG3-6 was comparable with the report of Yacob (2008) which is 0.00 and 0.09 for Afar and BHS sheep under station management conditions.

The estimates of h^2^t values for ADG0-3, ADG3-6, and ADG0-6 were 0.12, 0.11, and 0.023 respectively, which is in a similar range to the h^2^a estimates. The estimates were in the moderate range except for ADG0-6. Total heritability estimates for ADG0-3 and ADG0-6 were comparable with the finding of Abegaz (2002) for Horro sheep, which were 0.13 ± 0.04 and 0.04 ± 0.03, respectively.

## 6 Genetic parameter estimates for reproductive traits

The estimate of h^2^a and h^2^t for litter size were 0.28 ± 0.12 and 0.29, respectively (Table 4). The h^2^a estimate indicated that genetic improvement through direct selection for this trait would be high for Doyogena sheep. Compared with another study the current heritability estimate for litter size was higher. Abegaz (2002) estimated for litter size using direct additive and repeatability models were 0.15 and 0.07 respectively for Horro sheep. Matika (2003) and Mohammadi et al. (2012) reported estimated heritability of 0.26 for Sabi and 0.14 for Zandi sheep by fitting the threshold model. Haile et al. (2020) reported h^2^a of 0.08 ± 0.041 and 0.08 ± 0.04 for Bonga and Horro sheep breeds respectively under CBBP and that is lower than the current estimates. Likewise, Khan et al. (2017), and Mohammadabadi and Sattayimokhtari (2013) estimated litter size heritability of 0.25 and 0.06 ± 0.02 for Rambouillet and Kermani sheep breeds in India and Iran respectively and the h^2^ values reported were lower than the current finding.

**Table 4:** Co (variance) components and genetic parameter estimates for reproductive traits.

Parameter	Models of Estimation					
	1	2	3	4	5	6
**Litter size**						
${{\text{σ}}^{2}}_{a}$	0.09	**0.08**	0.070	0.084	0.037	0.084
${{\text{σ}}^{2}}_{c}$	-	**0.092**	-	-	0.090	0.090
${{\text{σ}}^{2}}_{m}$	-	**-**	0.012	0.044	0.037	0.044
${\text{σ}}_{am}$	-	**-**	-	-0.035	-	-0.035
${{\text{σ}}^{2}}_{e}$	0.19	**0.108**	0.20	0.155	0.110	0.009
${{\text{σ}}^{2}}_{p}$	0.28	**0.28**	0.28	0.24	0.28	0.24
$h^{2}a\pm S.E$	0.32 ± 0.12	**0.28 ± 0.12**	0.13 ± 0.15	0.33 ± 0.2	0.13 ± 0.15	0.33 ± 0.20
$c^{2}\pm S.E$	-	**0.31 ± 0.01**	-	-	0.33 ± 0.007	0.38 ± 0.02
$h^{2}m\pm S.E$	-	**-**	0.13 ± 0.1	0.18 ± 0.1	0.13 ± 0.10	0.24 ± 0.08
$r_{am}\pm SE$	-	**-**	-	-0.58 ± 0.23	-	-0.58 ± 0.23
h^2^t	0.32	**0.29**	0.27	0.30	0.20	0.30
**Lambing interval**						
${{\text{σ}}^{2}}_{a}$	**1248.3**	1218.7	1277.5	1379	1256.2	1319
${{\text{σ}}^{2}}_{c}$	**-**	37.64	-	-	173.38	2675
${{\text{σ}}^{2}}_{m}$	**-**	-	0.86	6.11	0.73	1.6
${\text{σ}}_{am}$	**-**	-	-	-72	-	-1.4
${{\text{σ}}^{2}}_{e}$	**4882.7**	4874.7	4856	4746	4704	2140
${{\text{σ}}^{2}}_{p}$	**6131**	6131	6134	6060	6134.3	6134
$h^{2}a\pm S.E$	**0.20 ± 0.5**	0.19 ± 0.51	0.20 ± 0.55	0.22 ± 0.05	0.20 ± 0.05	0.21 ± 0.56
$c^{2}\pm S.E$	**-**	0.06 ± 0.001	-	-	0.03 ± 0.002	0.43 ± 0.56
$h^{2}m\pm S.E$	**-**	-	0.001 ± 0.4	0.001 ± 0.1	0.0 ± 0.4	0.0 ± 0.8
$r_{am}\pm SE$	**-**	-	-	-0.78 ± 0.0	-	-0.03 ± 00
h^2^t	**0.20**	0.20	0.20	0.21	0.20	0.21
**Age at first lambing**						
${{\text{σ}}^{2}}_{a}$	**9.60**	0.78	6.14	3562	1724.9	2061.6
${{\text{σ}}^{2}}_{c}$	**-**	12183	-	-	12129	11208
${{\text{σ}}^{2}}_{m}$	**-**	-	156.39	18129	1.09	2831
${\text{σ}}_{am}$	**-**	-	-	-8036	-	-2414
${{\text{σ}}^{2}}_{e}$	**13572**	1541.5	13420	0.028	0.9	0.002
${{\text{σ}}^{2}}_{p}$	**135582**	13726	13582	13655	13896	13376
$h^{2}a\pm S.E$	**0.001 ± 0.3**	0.11 ± 0.45	0 ± 0.00	0.16 ± 0.76	0.12 ± 0.56	0.15 ± 0.7
$c^{2}\pm S.E$	**-**	0.83 ± 0.00	-	-	0.87 ± 0.156	0.81 ± 0.47
$h^{2}m\pm S.E$	**-**	-	0.012 ± 0.7	-	0.014 ± 0.17	0.20 ± 0.33
$r_{am}\pm SE$	**-**	-	-	-99 ± 0.02	-	-0.98
h^2^t	**0.0007**	0.0001	0.006	0.04	0.12	0.008

σ^2^a = direct additive genetic variance; σ^2^c = maternal permanent environmental variance; σ^2^m = maternal additive genetic variance; σ_am_ = additive and maternal additive genetic covariance, σ2e = residual variance, σ^2^p = phenotypic variance, h^2^a = direct heritability c^2^a = ratio of maternal permanent environmental variance to phenotypic variance, h^2^m = maternal heritability; r_am_ = correlation between direct maternal additive genetic effects, h^2^t = total heritability and SE = standard error.

The h^2^t of 0.29 means that 29% of the variability in this trait between the ewes is due to genetic differences among ewes. Therefore, the current estimate of heritability shown for litter size has the highest heritability range, and thus, genetic improvement through selection for this trait would be high. The estimate of maternal permanent environmental variance (c^2^) was 0.31 ± 0.01. The results indicated that the inclusion of maternal permanent environmental effects in the analyses could improve the models for litter size traits. The reflection of variance due to maternal permanent environmental effect indicates improving the environment could improve the reproductive performance of ewes.

Under the best-fitted model, the estimated h^2^a and h^2^t for LI were 0.20 ± 0.5 and 0.20 respectively. Lobo et al. (2009) and Abdoli et al. (2019) estimated h^2^a of 0.06 and 0.02 for LI for Brazilian multi-breed sheep and Iranian Lori-Bakhtiari sheep and the values reported were lower than the current estimate. The h^2^a and h^2^t estimate for AFL were 0.001 ± 0.32 and 0.007, respectively. Since AFL is strongly influenced by environmental effects, low heritability estimate was obtained. Selection based on AFL performance may result in slow genetic improvement. Therefore, selection for ewes for AFL trait of ewes should be based on female relatives of ewes or on correlated traits, which have high and positive genetic correlation with ewes AFL. Lobo et al. (2009) and Abdoli et al. (2019) estimated 0.04 and 0.07 of AFL h^2^a for Brazilian multibreed meat sheep and Lori-Bakhtiari sheep breeds respectively. The h^2^ values reported were higher than the current finding.

## 7 Correlation estimates between reproductive traits

The bivariate analysis of genetic correlation between reproductive traits is given in Table 5. Negative estimate of genetic correlation -0.44 ± 0.9 was obtained between LI and LS. However, the genetic correlation between AFL and LS was strong and negative (−0.98 ± 0.32). The present estimate of genetic correlations was similar to the other estimates reviewed by Safari et al. (2005). Khan (2017) also reported comparable genetic correlation of -0.00 ± 0.02 and -0.006 ± 0.02 for LS with AFL and LS with LI in Rambouillet Sheep respectively.

**Table 5:** Estimates of genetic (below diagonal) and phenotypic (above diagonal) correlations between reproductive traits in bivariate analysis.

Trait	Lambing interval	Litter size	AFL
Lambing interval	-	0.018 ± 0.04	_
Litter size	-0.44 ± 0.9	-	-0.13 ± 0.11
AFL	_	-0.98 ± 0.32	-

## 8 Genetic correlations between growth traits

The present study indicated that BWT had weak genetic correlation with the studied body weights and daily weight gain traits (Table 6). The negative correlation (−0.35 ± 0.14) between AD’G0-3 and ADG3-6 indicated that lambs that grew faster in the pre-weaning period, grew more slowly during post-weaning period and vice versa. Mohammadi et al. (2015) reported similar findings to the present study in the Lori sheep breed. A moderate positive genetic correlation was observed between WWT and SMWT (0.52 ± 0.09). The positive genetic correlations between the two traits indicated that the genes that are responsible for increasing WWT result in increasing in SMWT trait. It could be used as selection criteria for improvement in body weight traits. The positive and moderate genetic correlation that post-weaning body weights and body weight gains may be under the influence of the same set of genes (Pleiotropy). In the ongoing CBBP both WWT and SMWT traits were considered the most appropriate selection criteria (Jembere et al., 2016). The current genetic correlations were similar to the report of Abegaz (2002) estimated for Horro sheep.

**Table 6:** Estimates of genetic (below diagonal) and phenotypic (above diagonal) correlations between growth traits in multi-trait analysis.

Trait	BWT	WWT	SMWT	ADG0-3	ADG3-6	ADG0-6
BWT	-	0.23 ± 0.02	0.17 ± 0.02	-0.01 ± 0.23	0.005 ± 0.02	-0.01 ± 0.02
WWT	0.21 ± 0.07	-	0.35 ± 0.02	0.65 ± 0.01	-0.30 ± 0.02	0.22 ± 0.02
SMWT	0.21 ± 0.09	0.52 ± 0.09	-	0.19 ± 0.02	0.54 ± 0.02	0.74 ± 0.01
ADG0-3	-0.003 ± 0.09	0.95 ± 0.03	0.37 ± 0.12	-	-0.46 ± 0.02	0.33 ± 0.02
ADG3-6	0.06 ± 0.12	-0.23 ± 0.13	0.70 ± 0.09	-0.35 ± 0.14	-	0.67 ± 0.01
ADG0-6	0.06 ± 0.1	0.53 ± 0.12	0.97 ± 0.04	0.43 ± 0.13	0.66 ± 0.09	-

BWT = birth weight, WWT = weaning weight, SMWT = six-month weight, ADG0-3 = average daily gain from birth to weaning age, ADG3-6 = average daily gain from weaning to 6 months age, ADG0-6) = average daily gain from birth to 6 months age.

## 9 Growth traits genetic trends

### 9.1 Birth weight (BWT)

**Figure 2:**
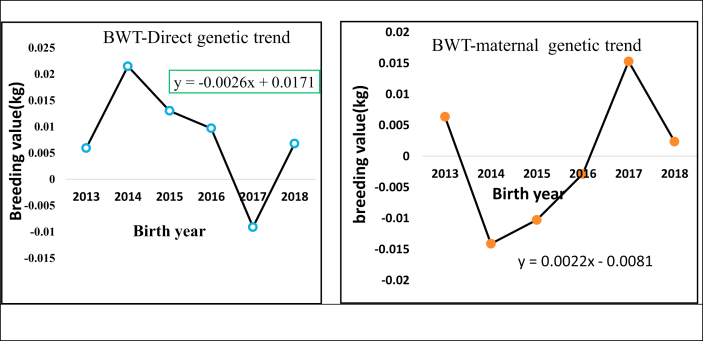
Birth weight direct genetic and maternal genetic trend.

Both direct genetic and maternal genetic trends had fluctuating trends (Figure 2). The annual estimated direct genetic trend was negative (−0.0026 kg/year) and insignificant (p > 0.05), however, the maternal genetic trends showed an increasing trend (0.0023 kg/year**.** The estimated direct and maternal genetic changes were 0.00085 kg and -0.004 kg respectively. When compared to other studies, Gizaw et al. (2014a) reported higher and positive genetic change in BWT for Menz sheep. The author reported a genetic gain of 0.005 kg in the 4th generation of selection program. Gholizadeh et al. (2015) reported an annual genetic gain of 0.00 kg/year for Baluchi sheep, which is almost similar to the current finding. Since direct genetic gain for the traits showed slightly negative trend, demonstrates that these traits should not be taken into consideration in the selection process by breeder cooperatives.

### 9.2 Weaning weight (WWT)

**Figure 3:**
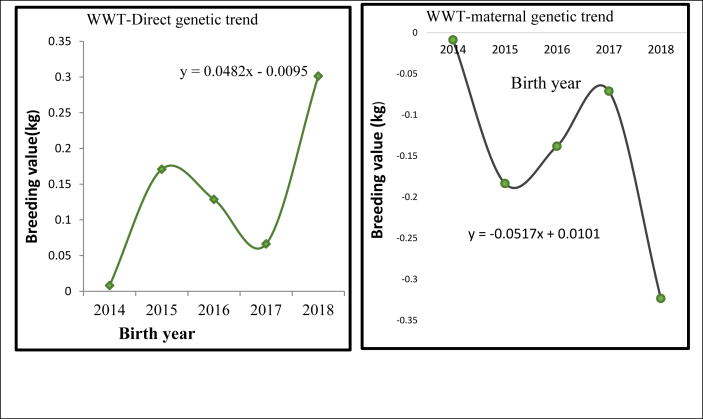
Weaning weight direct genetic and maternal genetic trend 6-month weight (SMWT).

Figure 3 shows the value of direct genetic and maternal genetic trends over the selection period. The magnitude of direct genetic trends estimated illustrates that there has been significant (p < 0.05) and positive genetic improvement in WWT with 0.3 kg in a period of 6-years selection (0.048 kg/year). From 2015 to 2017, the direct genetic trend was in decreasing trend, after which there was a slightly increased in values. The negative trend obtained could be due to the reason that less rainfall caused lack of forage and then decline in the growth performance of the flock within the selection years.

The maternal genetic trend had a decreasing trend by -0.051 kg/year and -0.23 kg per 6-years selection. The direct genetic trend of 0.048 kg/year was higher than the study of Mostafa et al. (2011) for the Arman sheep breed (0.007 kg/year) and lower than the report of Mokhtari (2010) for kermani sheep (0.125 kg/year). The direct genetic change (0.3 kg) was positive and significant for WWT. Gizaw et al.(2014a) reported 0.45 kg of genetic change in the 4^th^ generation of Menz sheep which is higher than the current finding.

**Figure 4:**
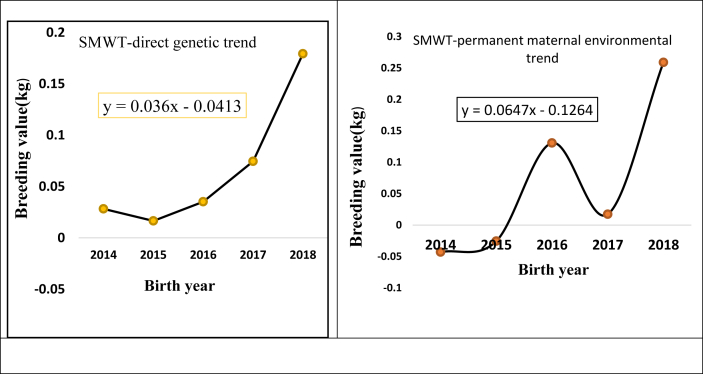
6-month weight direct genetic and permanent environmental trend.

In Figure 4 and Table, 7 annual genetic trend and genetic change for SMWT has been shown. The existing method of breeding ram selection was based on SMWT. The estimated annual direct genetic trend (0.036 kg/year) was positive and highly significant (p < 0.01). The fit of the regression shows 73.4% coefficient of determination with the regressed value. Direct genetic change was 0.15 kg. The estimate of direct genetic change (0.151 kg) for SMWT provides a good picture of the selection program concerning SMWT and therefore, continuation of selection based on SMWT trait is suggested to become more successful. The present estimate of direct genetic trend was concurrent with the study of Mokhtari and Rashidi (2010) for Kermani sheep (Mohammadi et al., 2011) and for Zandi sheep was 0.021 kg/year. Shaat (2004) and Arora et al. (2010) reported higher estimates in Rahmani sheep (0.135 kg/year) and for Malpura sheep (0.061 kg/year respectively. The present estimate is lower than the report of Gizaw et al. (2014a) in Menz sheep; they report 1.3 kg genetic progress at the 4^th^ generation.

**Table 7:** Estimates of genetic progress for growth traits in Doyogena sheep.

Trait	Annual direct genetic trend (kg/year)	Direct (kg)	Maternal (kg)	p-value	R^2^
BWT	y = -0.0026x + 0.0171	0.00085	-0.00401	0.09	0.23
WWT	y = 0.0482x - 0.0095	0.30	-0.23	0.014	0.46
SMWT	y = 0.036x - 0.0413	0.151	-	0.0002	0.73

## 10 Reproductive traits genetic trends

**Figure 5:**
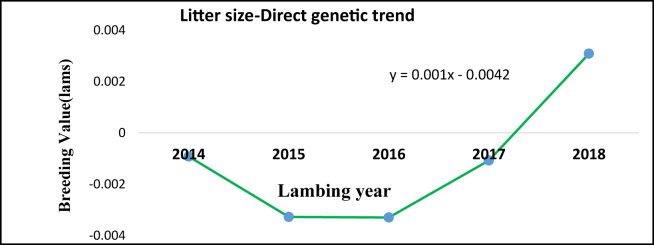
Overall cooperative litter size genetic progress.

Direct genetic trend for litter size was fluctuations (Figure 5) and non-significant (p > 0.05). This fluctuated and slow genetic trend, for litter size, could be because of lack of data quality. Genetic progress for LS showed improvement across selection years and could be taken into consideration in the process of selection.

**Figure 6:**
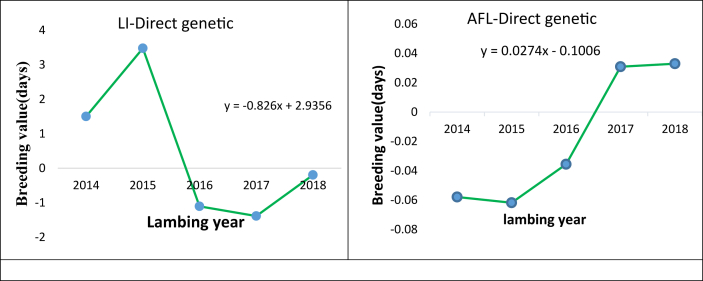
Lambing interval (Left) and age at first lambing (right) direct genetic trend.

LI shows (Figure 6) a decreasing trend (−0.0007 days/year). However, AFL trait showed an increasing trend (0.0174 days/year). Annual genetic changes in each of the reproductive traits considered in the present study are presented in Table 8.

**Table 8:** Estimates of reproductive traits genetic change.

Trait	Overall direct genetic	Maternal genetic	Environmental change	Annual direct genetic change	p-value	R^2^
Litter size	0.0004	-0.0009	≈0.00	y = 0.001x - 0.0042	0.7	0.38
LI	-1.69	-	-	y = -0.826x + 2.9356	0.18	0.42
AFL	0.09	-	-	y = 0.0274x - 0.1006	<0.001	0.85

## 11 Conclusion

The objective of this study was to evaluate community-based breeding program breed improvement strategy to further improve genetic gains in Doyogena sheep. Promising results of selection were observed from the ongoing Doyogena sheep CBBP. The different estimates of heritability obtained from the different models suggest that model choice is an important aspect of obtaining reliable parameter estimates to be used in genetic parameter estimation. The moderate to high estimated heritability for the growth traits suggested the scope for further improvement of these traits. Coefficient of inbreeding showed an increasing trend across selection years. There was a negative genetic trend observed for BWT trait. However, WWT and SMWT traits were genetically improved across the years. The estimate of genetic gain for SMWT trait was the greatest among the body weight traits. The result suggested that selection for litter size trait would be more efficient, due to its higher genetic variance; however, it may hurt growth performance.

## Declarations

### Author contribution statement

Kebede Habtegiorgis: Conceived and designed the experiments; Analyzed and interpreted the data; Wrote the paper.

Aynalem Haile; Tesfaye Getachew: Performed the experiments; Contributed reagents, materials, analysis tools or data; Wrote the paper.

Manzoor Ahmed Kirmani: Performed the experiments; Contributed reagents, materials, analysis tools or data.

Deribe Gemiyo: Contributed reagents, materials, analysis tools or data.

### Funding statement

This work was supported by the International Centre for Agricultural Research in the Dry Areas (ICARDA) and Southern Agricultural Research Institute (SARI).

### Data availability statement

Data will be made available on request.

### Declaration of interest’s statement

The authors declare no conflict of interest.

### Additional information

No additional information is available for this paper.
